# Proof of Concept for Prevention of Natural Colonization by Oral Needle-Free Administration of a Microparticle Vaccine

**DOI:** 10.3389/fimmu.2020.595320

**Published:** 2020-10-23

**Authors:** Rafael Frandoloso, Somshukla Chaudhuri, Gabriela Carolina Paraboni Frandoloso, Rong-hua Yu, Anthony Bernard Schryvers

**Affiliations:** ^1^Laboratory of Microbiology and Advanced Immunology, Faculty of Agronomy and Veterinary Medicine, University of Passo Fundo, Passo Fundo, Brazil; ^2^Department of Microbiology, Immunology & Infectious Diseases, Cumming School of Medicine, University of Calgary, Calgary, AB, Canada; ^3^AFK Imunotech, Passo Fundo, Brazil

**Keywords:** needle-free vaccination, mucosal immunization, colonization, Glässer’s disease, transferrin binding protein, microparticle vaccine, *Glaesserella parasuis*

## Abstract

There has been substantial interest in the development of needle-free vaccine administration that has led to a variety of approaches for delivery through the skin for induction of a systemic immune response. The mucosal administration of vaccines has inherently been needle-free, but the simple application of vaccines on the mucosal surface by itself does not lead to mucosal immunity. Since many important bacterial infections develop after initial colonization of the upper respiratory tract of the host, prevention of colonization could not only prevent infection but also eliminate the reservoir of pathogens that reside exclusively in that ecologic niche. This study was designed to provide proof of concept for a needle-free immunization approach that would reduce or eliminate colonization and prevent infection. In order to accomplish this a microparticle vaccine preparation was delivered just below the oral mucosal epithelial cell layer where it would lead to a robust immune response. A vaccine antigen (mutant transferrin binding protein B) shown to be capable of preventing infection in pigs was incorporated into a polyphosphazene microparticle preparation and delivered by a needle-free device to the oral sub-epithelial space of pigs. This vaccination regimen not only provided complete protection from infection after intranasal challenge by *Glaesserella parasuis* but also eliminated natural colonization by this bacterium. Notably, the complete prevention of natural colonization was dependent upon delivery of the microparticle preparation below the epithelial layer in the oral mucosa as intradermal or intramuscular delivery was not as effective at preventing natural colonization. This study also demonstrated that a primary immunization in the presence of maternal antibody limited the resulting antibody response but a robust antibody response after the second immunization indicated that maternal antibody did not prevent induction of B-cell memory.

## Introduction

Many important pathogens of humans and food production animals reside exclusively on the mucosal surfaces of the upper respiratory, oral or genitourinary tract where they serve as a reservoir for potential infection of their respective host. This includes Gram-negative bacteria in the Pasteurellaceae, Neisseriaceae, and Moraxellaceae families that possess surface receptors capable of acquiring iron directly from the host iron-binding proteins on the mucosal surface or, if they cross the epithelial cell barrier, within the body (1). Commercial vaccines developed to prevent infection have primarily been designed for parenteral (needle and syringe) administration thus, were not initially anticipated to influence colonization. However, studies have shown that the conjugate capsular vaccines targeting human pathogens responsible for meningitis and sepsis reduce colonization and ultimately eliminate strains expressing the targeted capsule types (2, 3). This provided the opportunity to achieve herd immunity, reducing disease by the targeted capsular types in non-vaccinated adult populations (4). The ability to impact colonization has not been observed for the protein components of the commercial meningococcal vaccine (5), suggesting that alternate routes of administration need to be considered for reducing or eliminating colonization.

Although there has been considerable effort at exploring vaccine approaches for human pathogens, they are normally focused on infection or colonization models in small animals or performing post-licensure studies with approved vaccines, both of which have major limitations. We opted to implement studies with the porcine pathogen *Glaesserella (*formerly *Haemophilus) parasuis* to provide proof of concept for a novel vaccine approach designed to achieve an immunological response that would be effective on the mucosal surface.

*Glaesserella parasuis* is a common inhabitant of the porcine upper respiratory tract, its only ecological niche, but may disseminate to distant systemic sites by entering into the bloodstream and produce a severe inflammatory disease in young piglets, known as Glässer’s disease (6, 7). Glässer’s disease can be prevented by the use of whole cell inactivated vaccines (bacterins) administered by the intramuscular route, which induces protective immunity through systemic IgGs capable of detecting and killing *G. parasuis* by classical complement system activation or opsonophagocytosis (8). Despite the availability and use of the bacterin vaccines, *G. parasuis* is present in many pig production facilities, particularly multi-site pig production systems where there is a risk of transmission between animals from different origins.

We initially targeted the transferrin receptors in *G. parasuis* based on the observation that they are present in all strains and were shown to be essential for disease causation in a porcine infection model with a closely related porcine pathogen *Actinobacillus pleuropneumoniae* (9). Initial attempts at preventing experimental Glässer’s disease with a vaccine formulated with native outer membrane vesicles from *G. parasuis* enriched by a transferrin-Sepharose column was effective whereas some recombinant sub-fragments of the wild-type transferrin binding proteins (Tbps) only provided partial protection (7). Capitalizing on the success in solving the structure of the surface lipoprotein, TbpB (10), a binding deficient mutant of TbpB (TbpB^Y167A^) was developed and shown to provide superior protection than the wild-type protein or a commercial vaccine preparation (11).

The demonstration that the diversity of TbpBs was distributed into three phylogenetic clusters that were not strongly associated with the time of isolation, geographical region or even species (present in *G. parasuis, Actinobacillus pleuropneumoniae* and *A. suis*) (12) suggested that it would be possible to design a TbpB-based vaccine effective against these three porcine pathogens. The TbpB^Y167A^ antigen was able to provide protection against strains expressing a homologous or heterologous TbpB from the same phylogenetic cluster (13) (11). These results suggest that it will be possible to generate complete protection against Glässer’s disease regardless of the capsular type of this bacterium and extend the protective effect to *Actinobacillus pleuropneumoniae* and *A. suis* due to the shared distribution of variants amongst these species (12).

In this study we have formulated a novel vaccine containing TbpB^Y167A^ for administration to the oral mucosa of pigs using a needle-free device. This vaccine induced mucosal and systemic antibody responses and protected immunized pigs during an experimental challenge by *G. parasuis*. In addition to protection against infection, the vaccine was able to prevent natural nasal colonization by *G. parasuis* in piglets reared in a pig farm with endemic circulation of *G. parasuis*. This result could serve as proof of concept for needle-free delivery of vaccines in humans, particularly since the device used in this study is designed for administration of anaesthetics in dental practice.

## Materials and Methods

### *G. parasuis* Strain and Growth Conditions

Growth of *G. parasuis* was as described previously (13). An aliquot of SV7 reference strain 174, which was previously passaged in pigs, was inoculated into supplemented PPLO broth [60 μg/ml nicotinamide adenine dinucleotide (*β*-NAD, Sigma-Adrich, USA) and 2.5 mg/ml D-glucose (Sigma-Aldrich, USA)] and grown with shaking (250 rpm/37°C) to achieve an optical density of 0.5 at 600 nm. The bacteria were collected by centrifugation, washed twice in DPBS (pH 7.2), and the bacterial concentration adjusted as required in a final volume of 0.5 ml of RPMI 1640 medium (Invitrogen, USA).

### Determination of the Intranasal Challenge Dose

In our previous studies *G. parasuis* Nagasaki (SV5) (7, 11) and *G. parasuis* 174 (SV7) (13) strains were used to challenge colostrum-deprived pigs by the intratracheal route. Here, we aimed to use the intranasal route to model a natural infection; hence two independent infection experiments were conducted with strain 174. In the first experiment, 12 piglets that were 28 days old were acquired from Swine Research and Technology Centre (SRTC) at the University of Alberta and transported to the Veterinary Science Research Station (VSRS) at the University of Calgary and randomly assigned to three different groups (four animals per group). After one week of acclimation, the animals were intramuscularly sedated with a drug cocktail composed of 20 μg/kg dexmedetomidine (Dexdomitor, Zoetis, Canada), 0.2 mg/kg azaperone (Stresnil, Vétoquinol, Canada), and 2 mg/kg alfaxalone (Alfaxan, Abbott, Canada) and challenged *via* the intranasal route with 2 × 10^6^ (group I), 4 × 10^7^ (group II), and 1 × 10^8^ (group III) colony forming units (CFU). A volume of 0.25 ml of the inoculum was dropped slowly in each nostril. Following the challenge, rectal temperatures and clinical symptoms such as joint swelling, lameness, dyspnea, tachypnea, coughing, sneezing and neurological signs were assessed at 12 h intervals until the experimental endpoint. When clinical symptoms were severe and the animals demonstrated signs of suffering, they were sedated and euthanized by intracardiac injection of 110 mg/kg of Euthanyl (Bimeda-MTC Animal Health INC, Canada). The second experiment was conducted to replicate the results observed in the first infection round. In this instance, 10 piglets obtained from the same provider at the same age were assigned equally in two groups (n = 5) and challenged by the same route with 2 × 10^6^ (group I) and 4 × 10^7^ (group II) CFU. The animals were monitored post challenge as described above.

### Antigen Production

The gene encoding the Y167A variant of the *G. parasuis* SV7 TbpB was cloned into a custom T7 expression vector containing an N-terminal polyhistidine tag and transformed into *Escherichia coli *strain ER2566 and induced for expression as previously described (13). Briefly, the cultures were grown overnight in autoinduction media, and the protein was produced under lac induction. The cultures were pelleted by centrifugation and subsequently lysed. The crude lysates from the cells expressing the recombinant proteins were collected and subjected to Ni-NTA chromatography followed by anion exchange with a Q-Sepharose column to purify the protein. The fractions from the Q-Sepharose column analyzed by SDS-PAGE as pure, were pooled and buffer exchanged into phosphate buffer saline (PBS) and filter sterilized (0.22 μm).

### Preparation of Vaccine Formulations

**Figure 1:**
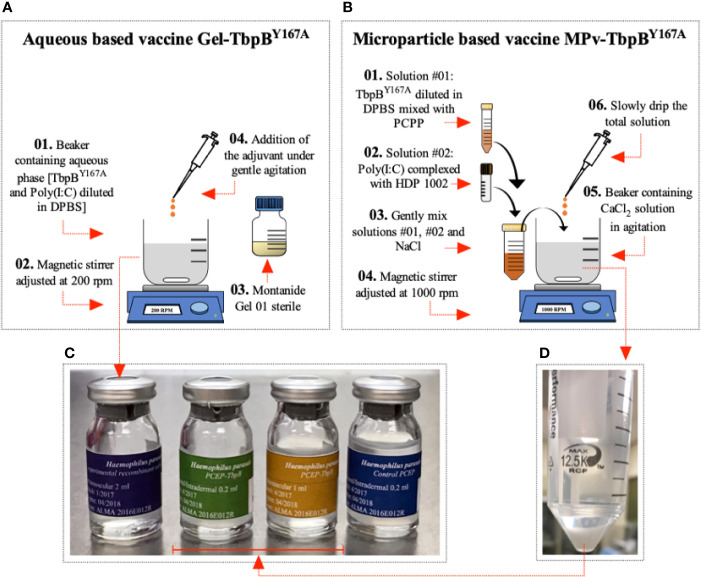
Preparation of vaccine formulations. **(A)** Preparation of the Montanide (Gel-TbpB ^Y167A^) vaccine formulation. (01) TbpB^Y167A^ and poly (I:C) was added to DPBS in a sterile beaker containing a magnetic stir bar. (02) The beaker was placed on a magnetic stirrer adjusted to 200 rpm and stirred for 10 min. (03) 20% (v/v) of the sterile Montanide Gel 01 adjuvant (04) was slowly added to the solution and stirred for 30 min. The product was kept overnight at 4°C. **(C)** The formulation was used to fill sterile glass tamper-proof bottles and sealed. **(B)** Preparation of the microparticle (Mpv-TbpB ^Y167A^) vaccine formulation. (01) TbpB^Y167A^ mixed with PCPP in DBPS and (02) poly (I:C) mixed with HDP in DPBS. (03) The two solutions were mixed with NaCl and (06) then slowly added to (05) a solution of CaCl_2_ (04) being stirred in a beaker at 1000 rpm. **(D)** The microparticles were collected and washed with DPBS, and then resuspended in DPBS and **(C)** used to fill sterile glass tamper-proof bottles and sealed.

The TbpB^Y167A^ microparticle based vaccine was designed to overcome the mucosal tolerance and induce a strong adaptive immune response. Microparticles (MPs) were generated by coacervation with sodium chloride (NaCl) and subsequent stabilization with calcium chloride (CaCl2) as described (14) with minor modifications (**Figure 1B**). Briefly, 10 ml Dulbecco’s PBS–DPBS (130 mM NaCl, 7.5 mM Na2HPO4, 1.5 mM KH2PO4, pH 7.4, Sigma-Aldrich, USA) containing 3 mg of TbpBY^167A^ was mixed with 225 μg of poly[di(carboxylatophenoxy)-phosphazene]—PCPP (Idaho Falls, ID, USA) dissolved in 500 μl of DPBS. The solution was incubated with gentle rotation at 22°C for 2 min followed by an additional 30 min incubation with no rotation. In parallel, 150 μg of low molecular weight poly (I:C) (InvivoGen, USA) was complexed with 300 μg of Host Defense Peptide (HDP) 1002 (VQRWLIVWRIRK-NH2, GenScript, Canada) at 37°C for 30 min. Subsequently, the two solutions were gently mixed by rotation and then 460 μl of 6.2% NaCl solution was slowly added followed by a 20 min incubation at 22°C without rotation. The total solution was then slowly added into a beaker containing 25 ml of 8.8% CaCl_2_ under high speed agitation (1,000 rpm) and was maintained under agitation for another 30 min. Finally, the microparticles were harvested by centrifugation (2,500 rpm for 10 min) and washed twice with 25 ml of DPBS (**Figure 1D**). DPBS was added to the final microparticle preparation pellet bringing the final volume to 3 ml and was distributed into sterile vials of the final preparation (**Figure 1C****)** referred to as the MPv-TbpB^Y167A^ vaccine formulation. All processes were carried out in a class II biosafety cabinet. The final formulation containing 200 μg TbpBY167A, 10 μg poly (I:C), and 20 μg HDP per 200 ul was used directly for oral and intradermal delivery or diluted to 1 ml with DPBS (pH 7.4) for intramuscular injection. The protein release assay (**Supplementary Figure 1**) demonstrated that this volume contained 200 μg of antigen.

The Gel-TbpB^Y167A^ vaccine was formulated by mixing 3 mg of TbpBY167A and 150 μg of poly (I:C) in DPSB with 20% (v/v) Montanide Gel 01 adjuvant (Seppic, France) to a final volume of 3 ml (**Figure 1A**) and then distributed aseptically into sterile glass tamper-proof bottles (**Figure 1C**). The bottles were closed to prevent contamination and labeled and maintained at 4°C until use. All vaccines used in this study were formulated once, avoiding any variation between different batches of the same formulation. Additionally, an adjuvant microparticle control formulation was prepared without the TbpB antigen but with the HDP and poly (I:C) in the composition (MPv-PBS). All procedures were conducted in a class II biosafety cabinet.

### Microparticle Assembly and Morphology Analysis

A refined confocal laser scanning microscope (CLSM) analysis was performed in order to characterize the microparticle assembly. Briefly, the MPs were prepared as described above using FITC-TbpB^Y167A^ (prepared following the FITC conjugation protocol suggested by Sigma-Aldrich, USA) and Rhodamine poly (I:C) (InvivoGen, USA). The MPs were analyzed under a Confocal Laser Scanning Microscope (CLSM) using the Olympus IX81 FV1000 Laser Scanning Confocal Microscope at the Live Cell Imaging Resource Laboratory (LCI) of the Snyder Institute at the University of Calgary. Images were captured and analyzed by ImageJ software. The establishment of the MPs size and morphology was conducted under a Scanning Electronic Microscope (SEM) using a FEI XL30 (Philips, USA).

### Release Kinetics of TbpB From Microparticles

Evaluation of the release kinetics of the TbpB antigen from the microparticle formulation was performed. Briefly, 6 mg of MPv-TbpB^Y167A^ was suspended in 6 ml of DPBS, aliquoted to 0.2 ml per tube, and incubated in a dark room under rotation at 39°C (simulating the physiological temperature of a piglet) for 30 days. The supernatant from the tubes were collected each day for 30 days and the quantity of the released TbpB was determined by spectrophotometry at 280 nm. To determine the total amount of TbpBY167A encapsulated prior to the start of the kinetic experiment, an initial aliquot was disrupted using 400 mM of EGTA (pH 7.6) and the supernatant quantified (**Supplementary Figure 1**). The experiment was conducted twice as two independent experiments.

### Vaccine Sterility

Sterility was assessed as recommended by the European Pharmacopeia. Soya-bean casein digest medium was used for the culture of both fungi and bacteria. Briefly, 1 ml of the vaccine preparation was inoculated in 9 ml of liquid medium and incubated for 14 days under shaking. As a positive control, one single colony of Staphylococcus aureus ATCC 6538 was inoculated in the mentioned medium. The presence of contaminant microorganism was determined by macroscopic turbidity and when no evidence of microbial growth was observed, the vaccine production was considered to be in compliance with sterility conditions.

### Safety and Immunogenicity Tests in Mice

In order to determine if the vaccines complied with the requirements for safety and immunogenicity, a total of twenty 7-week-old C57/BL6 mice were immunized twice, two weeks apart, with MPv-TbpBY167A (n = 10) and Gel-TbpBY167A (n = 10) *via* the subcutaneous route with the full dose of the pig vaccine designed for oral immunization (0.2 ml). Prior to each vaccination, 0.2 ml of peripheral blood was collected from the tail vein of each mouse to assess the antibody production against TbpBY167A. During the experiment, the site of injection and the animal behavior were monitored once a day.

### Immunization and Experimental Challenge of Pigs

**Figure 2:**
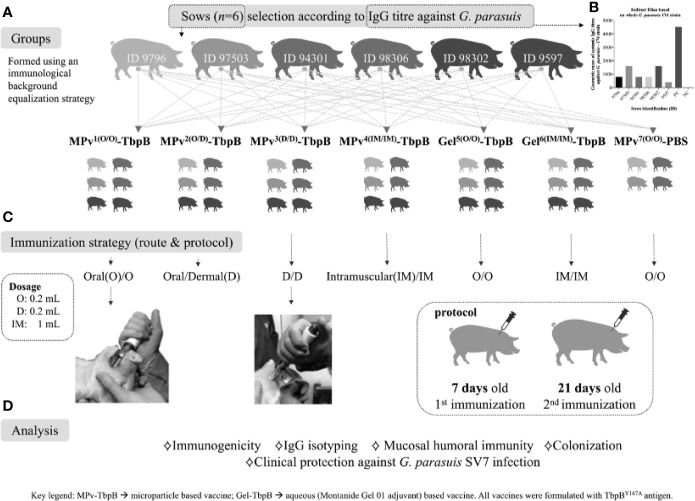
Graphic representation of the immunization and challenge experiment, including the immunological and bacteriological analyses performed. **(A)** Strategy for formation of groups using piglets from different sows to normalize the genetic and immunological background between groups. **(B)** Illustration of antibody titers against G. parasuis of the sows selected for this study. **(C)** Immunization schedule: piglets were vaccinated at 7 days old and revaccinated at 21 days old. The vaccination was performed by a direct intraoral or dermal administration using a needl- free device or by conventional intramuscular administration. **(D)** Description of the analyses conducted in this study. Mpv-TbpB -microparticle based vaccine, Gel-TbpB aqueous (Montanide Gel 01 adjuvant) based vaccine. TbpB – TbpB^Y167A^.

Forty commercial-hybrid Large White × Landrace pigs were used in this experiment. The piglets were born from six different sows serologically positive for *Glaesserella parasuis* as shown in **Figures 2A, B**. All sows were housed in the Swine Research and Technology Centre at the University of Alberta. Seven days after birth (D7); the piglets were randomly assigned in seven groups named MPv1-4-TbpB, [n = 24, body weight (BW) 2.6 kg ± 0.3] Gel5-6-TbpB [n = 12, body weight (BW) 2.6 kg ± 0.2] and Control (n = 4, BW 2.7 kg ± 0.2) and immunized using a new strategy of antigen delivery (**Figure 2C**). One piglet from group Gel6 died between the first and second immunizations from causes unrelated to this experiment. Mucosal immunizations were performed directly on the oral mucosa (jugal position) using a needle free device (Comfort-in™ Needle Free Injector Kit, Mika Medical CO, Korea). Briefly, the needle free syringe with a flat positioning cap was connected to the injector, pressurized, and then a dose of 0.2 ml of the MPv-TbpBY167A, Gel-TbpB or adjuvant control (MPv-PBS) vaccine was injected in the oral subepithelial space in animals of the groups MPv1and 2-TbpB, Gel5-TbpB and MPv7-PBS (control), respectively. Pigs from the groups MPv2 and 3-TbpB were immunized by intradermal route (0.2 ml) in the neck near the ear. Pigs from the groups MPv4-TbpB and Gel6-TbpB received an intramuscular injection (1 ml) in the neck. After the first immunization all piglets were returned to their sows until they became 21 days old (D21), at which point, a second vaccination was conducted. The piglets were weaned and transported to VSRS of the University of Calgary at 28 days old (D28). The intranasal challenge was performed 21 days (D42) after the second vaccination using 4 × 107 CFU as described above. Following the challenge, the clinical evaluation was conducted as described above. To assess the systemic and mucosal (oral) antibody titers, blood samples and oral swabs were collected at D7, D21, and D35. Nasal swabs were collected on D35 (7 days before challenge) and before necropsy.

### Molecular Detection of *G. parasuis* From the Upper Respiratory Tract

Nasal swabs (FLOQSwabs, Copan, Italy) for detection of *G. parasuis* were sampled at D35. The swabs were introduced deep in the two nostrils and rotated several times and then dipped in eppendorf tubes containing 250 μl of PBS. After vigorously vortexing the tubes, the total genomic DNA was extracted using MagaZorb® DNA Mini-Prep Kit (Promega, USA) following the manufacturer’s instructions. The presence or absence of *G. parasuis* was then determined by Real Time PCR (15).

### Antibody Analysis

To quantify the antibody production during the immunization process, pig antisera and oral fluid (oral swab) from all groups were analyzed in an indirect ELISA using streptavidin plates (ThermoFisher Scientific, USA) coated with biotinylated TbpBY167A. Blood samples for quantifying total IgG, IgG1, IgG2, IgM, and IgA were collected at D7, D21, and D35. Oral swabs to quantify secretory IgA were collected at D7, D21, and D35. Nasal swabs were collected at D35 and before necropsy to quantify secretory IgA. Sera and oral fluid were serially diluted from 1:100 to 1:51,200 and 0 to 1:8, respectively. Nasal swab washes were serially diluted from undiluted to 1:2,048. Briefly, 100 μl of diluted samples in PBST (PBS 0.05% Tween 20) containing 1% skim milk was added to the wells and incubated for 1 h at 37°C. The wells were washed five times with PBST and 100 μl of goat anti-pig whole IgG or anti-pig IgM peroxidase conjugated (all purchased from Sigma-Aldrich, USA) diluted at 1:5,000 were added to the wells and incubated for 1 h at 37°C before washing another five times with PBST. To determine the titers of IgG1, IgG2, IgA or secretory IgA, 100 μl of mouse anti-pig IgG1, IgG2, IgA or IgA secretory component (all purchased from Bio-Rad, USA) diluted at 1:2,500 was added to the wells and incubated for 1 h at 37°C. After washing the wells five times, 100 μl of goat anti-mouse IgG Fab with peroxidase conjugated (SouthernBiotech, USA) diluted at 1:5,000 was added to the wells and incubated for 1 h at 37°C, and the wells were washed again. The enzymatic reaction was developed with 3,3,5,5′-tetramethylbenzidine (Sigma-Aldrich, USA) + 0.06% H_2_O_2_ (Sigma-Aldrich, USA), and the plates were incubated in the dark at 22°C for 15 min before 3 N HCl was added stopping the reaction Plates were read at 450 nm using a Multiskan FC Microplate Photometer (ThermoFisher, USA). The results of the quantitative analysis were described as endpoint titers, which are the reciprocal of the highest dilution that gave a positive OD reading defined as at least two times greater that the OD values of the negative at 1:100 dilution.

### Necropsy, Sampling, and Bacteria Growth

Necropsy was immediately performed and documented following the death of an animal. The carcass was laid in dorsal decubitus and the members were rebated. Sterile incisions in the abdominal and thoracic cavities were made for bacteriological sampling, followed by incisions in the pericardium, central nervous system and articulations. The snout was transversally cut exposing the maxillary sinus for sampling. After sampling, the ribs and sternum were removed to expose the lungs and heart and abdominal cavity. The organs were removed, first the abdominal organs followed by the thoracic organs. The head skin was removed exposing frontal, parietal, and occipital bones; a hacksaw was sterilized and used to crack the bones in a triangle incision through frontal and lateral borders. After removing the skullcap, an incision was made through the meninges with a sterile scalp blade to collect swabs for bacteriological assay. Macroscopic lesions were scored as none, mild, moderate or severe. Swab samples taken during necropsy were seeded in chocolate agar plates and incubated at 37°C under 5% CO2 for 24–48h. G. *parasuis* colonies were molecularly confirmed by multiplex PCR (16).

### Statement of Institutional Animal Care

All animal experiments were carried out in accordance with the Guidelines for the Care and Use of Laboratory Animals as indicated by the Canadian Council on Animal Care and the University of Calgary (Protocol AC17-0153).

### Statistical Analysis

All data were analyzed using GraphPad Prism™ (GraphPad Software, San Diego, California, USA). The mean immunoglobulin tites from ELISAs were examined for significance using Two Way ANOVA with Sidak’s multiple comparisons test. The comparative survival analysis was performed by Kaplan–Meier curve analysis. The serological results are reported as geometric means ± 95% CI, and P-values < 0.05 were considered to be significant.

## Results

In order to implement our experiment to test the efficacy of needle-free delivery of a microparticle vaccine preparation on inducing mucosal immunity in pigs under conditions found in commercial production facilities, the study was performed at two academic institutions; the Swine Research and Technology Centre at the University of Alberta (U of A) and the Spy Hill Campus at the University of Calgary (U of C). The immunizations and samplings were performed at the U of A pig production facility that had endemic circulation of *G. parasuis* common to many commercial operations. After weaning, the tagged piglets were subsequently transferred to the U of C research facility for the challenge experiment.

Since our plans were to perform immunizations on days 7 and 21 after birth (**Figure 2**) it was important to consider the impact of maternal antibody acquired during suckling, that occurs efficiently until around day 3 (17). Sows were screened for their antibody levels against the *G. parasuis* challenge strain and for their litter sizes to select six sows that would readily supply the 40 piglets required for the planned experimental groups. The groups were designed to be comparable with respect to the maternal genetic background, maternal antibody and body weight (**Figure 2**). The experiment was also designed to compare different routes of administration of the microparticle formulation (MPv-TbpB) as well as a conventional recombinant-subunit formulation (Gel-TbpB) and a control microparticle preparation lacking antigen (MPv-PBS). Serum and swab samples were taken throughout the immunization period prior to challenge to analyze the immune response and evaluate the colonization status.

### Preparation of the Vaccine Formulations

**Figure 3:**
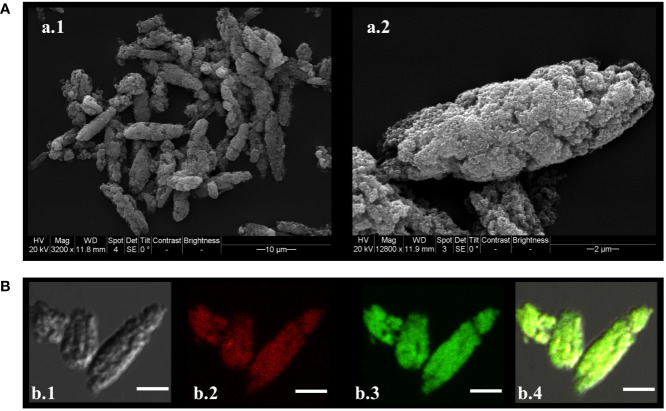
Microparticle morphology, size and assembly. **(A)** Scanning electron micrograph of MPv-TbpB^Y167A^. A.1) MPs observed at 3,200 times magnification; **(A)** 2) MPs observed at 18,000 times magnification. **(B)** Confocal laser scanning microscopic images (CLSM) of FITC-TbpB^Y167A^/HDP-Rhodamine-poly (I:C) MP. Maximum intensity projections of three slices from the Z-stacks (0.3 µm/slice) were created. B.1) Differential interference contrast (DIC) of the MP. B.2) HDP-Rhodamine-poly (I:C) layer localized under the external structure of MP. B.3) FITC-TbpB^Y167A^ observed in the core of the MPs. B.4) Merged DIC, HDP-Rhodamine-poly (I:C) (red) and FITC-TbpB^Y167A^ (green) images. Images were processed using Image J/Fiji (20, 21).

Our plan was to use a microparticle formulation to efficiently deliver antigen to the sub-epithelial space based on the rationale that it would overcome mucosal tolerance and induce a strong adaptive immune response. Initially methods for production of two types of microparticles were tested (18, 19) to evaluate which method was most effective at encapsulation of the protein antigen. The preliminary results (not shown) indicated that the polyphosphazene based microparticles were most effective at trapping the protein, so we implemented and slightly modified the published methods for their preparation (14). The microparticles had a spheroid morphology ranging in size between 10 and 20 μm (**Figure 3A**) with a rough surface. Confocal imaging analysis of the microparticles prepared with fluorescein-labeled TbpBY167A and rhodamine labeled poly (I:C) showed that TbpBY167A is localized in the core of the microparticles with the poly (I:C) forming an external and adjacent layer that is encapsulated by the polymer (**Figure 3B**).

A single batch of microparticle vaccine formulation (MPv-TbpBY167A) was prepared for consistency throughout the experiments. In an attempt to evaluate the potential release kinetics upon injection, the *in vitro* kinetics of TbpBY167A release from the microparticles (MPv-TbpBY167A) were assessed by spectrophotometry (**Supplementary Figure 1**). The increase in absorbance at 280 nm in the supernatant was used to measure the increase in release of TbpBY167A from the microparticles. The microparticles were incubated at 39°C, which is close to the physiological temperature of 21–35 day old piglets. TbpBY167A had an initial slow rate of release from the microparticles resulting in only 12% of the protein being released by day 6. This was followed by an increased rate of release so that only 10% of the protein remained in the microparticles by day 14. Aliquots of a control microparticle preparation kept at 4°C were treated with 400 mM of EGTA (pH 7.6) to provide a measure of the total total amount of TbpBY167A encapsulated in the MPv-TbpBY167A at each time point (**Supplementary Figure 1**).

A conventional subunit-recombinant vaccine formulation prepared with Montanide Gel 01 containing poly (I:C) (Gel-TbpBY167A), the microparticle preparation (MPv-TbpB) and a control microparticle vaccine formulation without antigen (MPv) were evaluated for sterility, safety, and immunogenicity. The vaccines were shown to be sterile, safe, and highly immunogenic in mice (**Supplementary Figure 2**).

### Optimizing Needle-Free Delivery

The rationale for oral delivery of the microparticles by a needle-free device is that it would be possible to deliver the preparation just below the epithelial cell layer where dendritic cells and other immune cells would facilitate the antigen uptake, processing, and presentation at the local lymph nodes. The ability to obtain fresh pig heads from cadavers provided the opportunity to evaluate the settings of the needle-free device that would deliver the microparticles to the most appropriate region. Since the soluble microparticles were unlikely to substantially alter the delivery of liquid droplets by the biolistic injector (Comfort-in™ applicator, needle-free injection system), we used a solution containing Coomassie blue dye and varied the settings [spacers with different thicknesses (2, 4, and 6 mm) which increase or decrease the pressure on the mucosa] for delivery of the liquid droplets. Subsequent macroscopic sectioning of the cadaver jugal mucosa showed that with a 2 mm rubber spacer we were able to deposit the total volume injected (0.2 ml) between the mucosal and muscular layer (results not shown).

### Development of an Intranasal Challenge Model

Since our goal was to evaluate the vaccine formulation’s ability to prevent natural infection by *G. parasuis*, we sought to implement an infection model that more closely mimics the natural infection process. Over the last decade we have performed *G. parasuis* challenge experiments in pigs using the intratracheal route since it delivers a consistent challenge dose to the lungs (6, 7, 11) and strategically overcomes the direct attack of the humoral and cellular components of the innate immune system present on the upper respiratory tract. To provide a more natural route of infection for vaccine evaluation we developed an intranasal infection model to mimic acquisition of *G. parasuis* by natural transmission.

**Figure 4:**
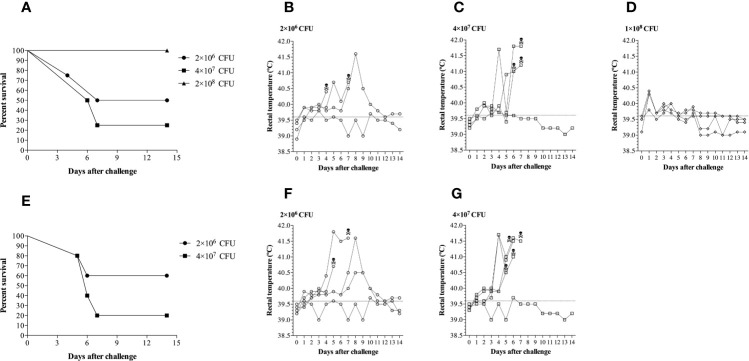
Clinical survival rates and rectal temperatures of pigs challenged with different doses of Glaeserella parasuis 174 strain by the intranasal route. **(A)** Evaluation of three doses of G. parasuis 174 strain [2×10^6^ (n=4), 4×10^7^ (n=4) and 1×10^8^ (n=4)] in conventional pigs. **(B–D)** Represent the rectal temperatures of each group infected with the doses described above. **(E)** A second challenge experiment to reproduce the results illustrated in the graphic 2a. Two doses of G. parasuis 174 strain [2×10^6^ (n=5) and 4×10^7^ (n=5)] were evaluated in conventional pigs. **(F, G)** Represent the rectal temperatures of each group infected with the two doses described above. After the challenge the pigs were monitored for clinical signs and symptoms throughout the duration of the experiment, which was 14 days. The skull represents the time of death.

In the first experiment (**Figures 4A–D**), three different challenge doses of *G. parasuis* 174 strain (SV7) were administered intranasally to groups of four pigs. Unexpectedly, the highest dose was not the most effective in achieving disease. Animals challenged with 1 × 10^8^ CFU of *G. parasuis* displayed some minor clinical signs (apathy) of disease but had a 100% survival rate until the end of the experiment (**Figure 4A**). Notably, three of these pigs had a higher rectal temperature on day 1 (**Figure 4D**) than the pigs from the other groups (**Figures 4B, C**) suggesting a strong activation of the innate immune response to the challenge. In contrast, the pigs infected with 2 × 10^6^ and 4 × 10^7^ CFU had mortality percentages of 50% (2/4) and 75% (3/4), respectively and had delayed and higher spikes of temperature in pigs with substantial clinical signs of disease (apathy, lameness, joint swelling, sneezing, coughing, dyspnea and neurological signs). The pathological lesions observed during the necropsy are provided in **Supplementary Table 1**.

To verify whether the results observed in the first experiment were reproducible, a second experimental challenge was conducted in two groups of five pigs using the doses of 2 × 10^6^ and 4 × 10^7^ CFU. As shown in **Figure 4E**, the dose of 4 × 10^7^ CFU was enough to trigger Glässer’s disease that resulted in death in 80% of the challenged pigs (4/5). In contrast, only 40% (2/5) of the pigs challenged with 1 × 10^6^ CFU died after challenge. The clinical course was very similar to the first experiment as was the pattern of the rectal temperatures (**Figures 4F, G****)**. These results indicate that there is a relatively narrow but reproducible window for an effective challenge dose in the intranasal model of infection.

### The Impact of Vaccination on Prevention of Natural Colonization

**Figure 5:**
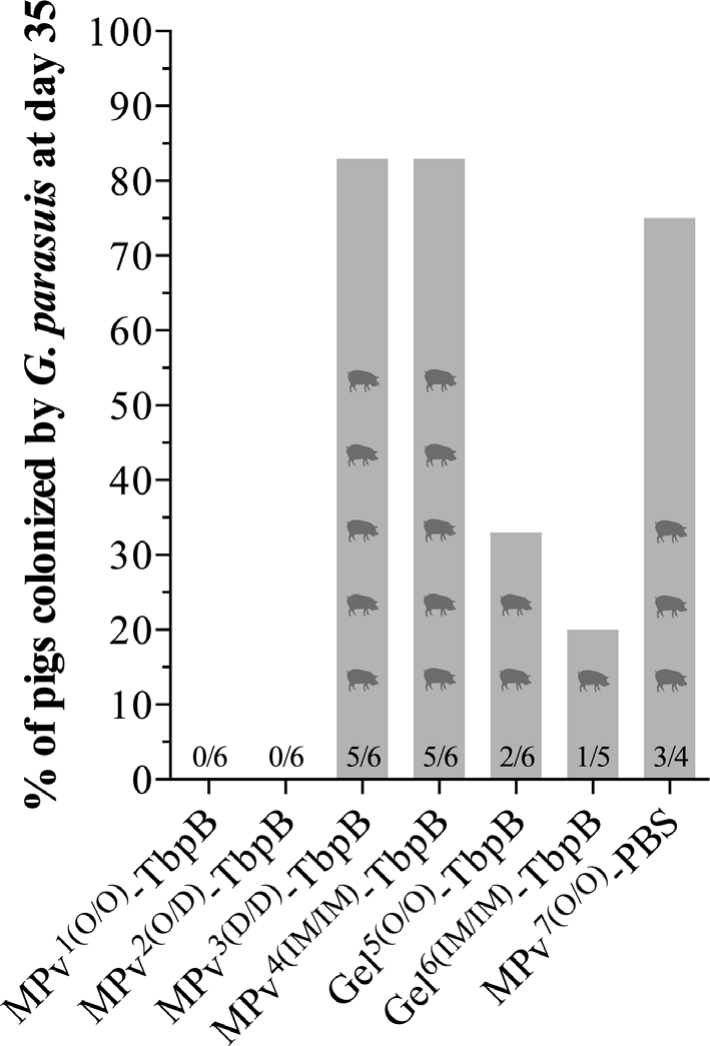
*Glaesserella parasuis* colonization affected by vaccination using MPv or Gel-TbpB based vaccines. Prior to the experimental challenge (D35), pigs were nasal sampled and *G. parasuis* DNA assessed by qPCR.

Our overall plan (**Figure 2**) included nasal sampling at day 35 of the experiment, seven days prior to the bacterial challenge. This allowed us to assess the ability of the vaccine formulations to prevent natural colonization of the upper respiratory tract by *G. parasuis* and also be able to consider what impact colonization might have on the antibody responses to be measured. Notably all pigs with a primary needle-free oral administration with the MPv- TbpB formulation (MPv1 and 2 TbpB) were negative for *G. parasuis* seven days before the challenge (14 days after the second immunization) (**Figure 5**). In contrast, most of the pigs (83%) immunized with the microparticle vaccine formulation by the dermal or intramuscular route were colonized by *G. parasuis* at D35 as were the adjuvant control group (75%) (**Figure 5**). The pigs immunized with Montanide Gel 01 formulation had lower colonization rates than the control and unexpectedly a higher colonization rate was observed for the formulation administered by the oral route (33%) than pigs immunized by the intramuscular route (**Figure 5**). It is important to note that the pigs in the various treatment groups were mixed throughout the entire experiment, thus the source of *G. parasuis* could not only be from the sow prior to weaning, but from the other piglets as the overall colonization rate at day 35 was 41%.

### The Impact of Vaccination on Serum, Oral, and Nasal Anti-TbpB Antibody Levels

**Figure 6:**
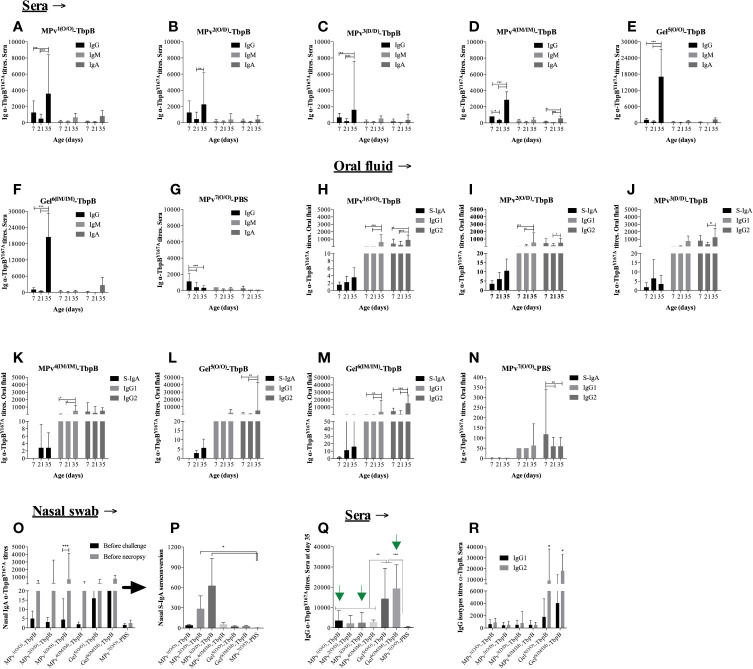
Immunoglobulin (Ig) profile induced by MPv-TbpB or Gel-TbpB vaccines. Systemic classes of *α*-TbpB^Y167A^ Igs (IgG, IgM and IgA) are illustrated in the graphics **(A–G)**. Secretory IgA, IgG1 and IgG2 assessed in the oral fluid are represented in the graphic **(H–N)**. Secretory IgA assessed in the nasal mucosal are illustrated in the graphic **(O, P)**. Comparison of total IgG titers its subclasses (IgG1 and IgG2) induced by vaccines before the experimental challenge are shown in the graphic **(Q, R)**, respectively. The results are expressed as geometric mean + 95% CI of the titers. Asterisks indicate significant differences (*p < 0.05, **p < 0.01 and ***p < 0.001) between the different time points.

As illustrated in **Figure 1** piglets were immunized at 7 and 21 days of life with either the MPv-TbpB formulation (n = 6), the Gel-TbpB formulation (n = 6) or the adjuvant control (n = 4) followed by challenge at day 42. Serum and oral swab samples were collected on day 7, day 21, and day 35 (7 days prior to challenge) whereas nasal swabs were collected on day 35 and before necropsy. Vaccinated and control pigs were assessed for anti-TbpBY167A titers in oral fluid and serum using an indirect in-house ELISA (**Figure 6**).

On day 21, 14 days after the first vaccination, a trend of decreasing anti-TbpB serum immunoglobulin (Ig) titers was observed for all analyzed classes or sub-classes of Ig in all animals (**Figures 6A–G**). As illustrated by the control adjuvant preparation (**Figure 6G**), this phenomenon can be attributed to the reduction of maternal antibody titers acquired by the piglets through colostrum ingestion. All sows were serologically positive for *G. parasuis* prior to parturition and all the pigs from the control adjuvant group displayed the same trend. In spite of the presence of antibody against TbpB during the first immunization, the second immunization on day 21 for all vaccines was immunogenic as all vaccinated animals displayed a significant increase of systemic IgGs on day 35, two weeks after the second immunization that was performed on day 21 (**Figures 6A–F**). Notably, the antibody titers induced by the Montanide Gel formulation were substantially higher than by the microparticle formulation regardless of the route of immunization (compare 6a to 6e and 6d to 6f). Animals from groups MPv1 and 2-TbpB and Gel6-TbpB had a boost of both IgG1 and IgG2 titers in the oral fluid after revaccination (**Figures 6H, I, M**) whereas animals from MPv3-TbpB and Gel5-TbpB groups had only their IgG2 titers increased (**Figures 6J, L**). Animals from MPv4-TbpB group had their IgG1 titers increased after revaccination (**Figure 6K**). Overall, the type of formulation and the delivery route of antigens had an influence on the modulation of the type of Ig associated within the oral mucosa. There was a biological increase, but not a statistically significant increase, in the secretory IgA titers in the oral fluid in the vaccine formulation groups but not the control group (**Figures 6H–N**).

As expected, all vaccinated pigs harbored significantly higher levels of anti (*α*)-TbpBY167A antibodies in the sera or oral fluid prior to challenge compared to the control pigs (MPv7-PBS) (**Figure 6**). Notably nasal secretory (S)-IgA was biologically elevated 7 days before the experimental challenge (D35) in vaccinated animals. Interesting, prior to necropsy we observed an increase of S-IgA compared to the D35 titers particularly in the pigs from the MPv1-4-TbpB groups (**Figure 6O**). The seroconversion level of nasal S-IgA was higher in pigs from MPv2 and 3-TbpB groups than pigs from the other groups and statistically different when compared with control group (**Figure 6P**). Taken together these results suggest that the MPv-TbpB formulation is able to induce a specific B cell response (Ig-secretory and memory cells) at the entry point of *G. parasuis* infection.

### The Impact of Vaccination on Prevention of Infection

**Figure 7:**
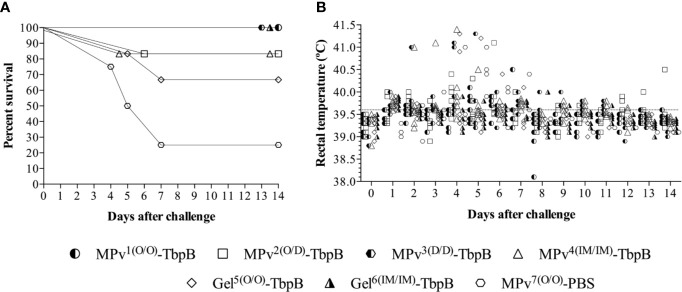
Clinical survival rates of immunized and challenged pigs. **(A)** Percent survival of the pigs challenged with 4 × 10^7^ CFU of G. *parasuis* by the intranasal route. The vaccine formulations and route of delivery are as indicated in **Figure 1**. MPv^1^-TbpB (Oral/Oral), MPv^2^-TbpB (Oral/Dermal), MPv^3^-TbpB (Dermal/Dermal), MPv^4^-TbpB (IM/IM), Gel^5^-TbpB (Oral/Oral), Gel^6^-TbpB (IM/IM). **(B)** Rectal temperature recording of the pigs after challenge.

No local or systemic adverse reactions were observed during daily inspections for two weeks following immunizations. Significant clinical signs (apathy, anorexia, sneezing, coughing, dyspnea, and joint swelling) were observed between days 2 and 9 after challenge in three of the four pigs in the control group but not in any of the pigs immunized with the MPv-TbpB vaccine administered with the needle-free device by the oral (O/O) or dermal (D/D) routes. As illustrated in **Figure 7**, all pigs immunized with formulations containing TbpBY167A were protected to varying degrees against the challenge whereas 75% of the control group animals died from Glässer’s disease. Notably the microparticle vaccine administered by the oral route and the Montanide Gel vaccine administered by the intramuscular route were fully protected but there was a trend for reduced efficacy when administered by the other routes. Comparison of the serum antibody levels against TbpBY167A (**Figure 6Q**) demonstrated that full protection (green arrows) was not determined by the titer of serum antibodies or IgG specific isotype polarization (**Figure 6R**), particularly for the Montanide-Gel 01 formulation delivered by the needle-free oral route which was the least protective. The macroscopic lesions observed during necropsy are described in **Supplementary Table 2**. Little to no lesions were observed in the groups MPv1-TbpB and Gel6-TbpB compared to the control group during necropsy.

Taken together the results in **Figures 5****–****7** suggest that the needle-free delivery of the microparticle formulation below the epithelial cell layer of the oral mucosa induces an immune response that prevents colonization which is not dependent upon induction of high serum IgG levels. The results also indicate that induction of sufficiently high levels of serum antibody by alternate formulations and routes can also impact natural colonization. It will be important to determine whether the ability to induce prevention of colonization by either or both mechanisms can be achieved by targeting other surface antigens.

## Discussion

Vaccines have played a major role in the control of infectious disease in humans and animals. The delivery of vaccines has most commonly been achieved through the use of needles and syringes, which are undoubtedly effective, but do have limitations such as needle-stick injuries, disease transmission from improper use, and, in humans, reluctance due to needle phobia (22). In 2003, WHO reported on the burden of diseases from needle-stick injuries (NSIs) in health care workers (HCWs), which showed that there were 3 million accidental NSIs leading to 37% of all new hepatitis B cases in HCWs, 39% of new hepatitis C cases, and approximately 5.5% of new HIV cases (23). This has led to a recognized need for the development of needle-free vaccination approaches that can be applied to global immunization efforts, notably recognized as a Grand Challenge in Global Health in 2003 (24, 25).

Needle-free delivery through the skin has the potential of delivering the vaccine formulation to tissue that has significant numbers of dermal dendritic cells that are thought to be involved in the immune response along with other cell types to invoke a strong immune response, thus may require lower doses of antigen (22, 26). For dermal immunization, topical application approaches require specialized vaccine preparations such as inclusion of bacterial toxins (27) whereas jet injection methods can be used for many off-the-shelf vaccine formulations (22). The value and utility of particle or liquid injection devices have not been fully realized and although some trials have demonstrated they are equivalent in efficacy to the standard needle and syringe immunizations, there were actually more injection site reactions (28).

For veterinary applications needle-free delivery systems have the potential to increase the efficiency and reduce the cost of vaccination providing that the upfront costs for the needle-free device can be mitigated by the extent of use and lifetime consumption (29). Importantly, due to the physical characteristic (reduced volume) of the vaccine formulations designed to be delivered by needle-free devices, the incidence of local reaction (granuloma or abscess formation) at the vaccine injection site can be drastically diminished; and mitigate the economic losses derived from abnormal pork meat associated with intramuscular vaccination, which represent more than $1.7 USD per slaughtered pig in the case of animals vaccinated for foot-and-mouth disease (30). Clearly increasing labor costs could readily change the equation which suggests that there will be increasing usage of needle-free devices over time.

Mucosal immunization is inherently needle-free, and a variety of different approaches have been developed for delivery to the different mucosal surfaces in the body (22). Immunization of the nasal mucosa by sprays or immunization of the pulmonary mucosa by aerosol delivery has the potential for inducing a local mucosal immune response that could impact pathogens colonizing the respiratory tract prior to or during the pathogenesis of infection. However, the mucosal surface harbors complex microbial communities and is exposed to many materials that do not pose a risk of infection thus simple exposure on the mucosal surface does not by itself induce an effective immune response. Thus, aerosols containing protein antigens delivered to the nasal or pulmonary mucosa require adjuvants or components that facilitate their uptake across the epithelial layer in order to induce a protective immune response. To the best of our knowledge this is the first study to provide proof of principle for a needle-free device delivering a vaccine formulation across the epithelial layer of a mucosal surface and inducing an effective immune response against infection and colonization.

The most notable observation from this study is that delivery of the microparticle preparation below the epithelial layer not only prevented the development of infection but eliminated natural colonization by *G. parasuis* (**Figure 5**). For bacterial pathogens like *G. parasuis* that exclusively reside in the upper respiratory tract of their mammalian host this can result in elimination of bacteria that express the targeted antigen from the population. For example, the implementation of conjugate capsular vaccines for *Streptococcus pneumomiae* resulted in eventual elimination of strains expressing the targeted capsular types not only from the children that were immunized (2) but also resulted in reduction of disease by the targeted capsular types in the elderly unvaccinated (4). Similarly, the vaccine targeting strains of *Neisseria meningitidis* expressing the group C capsular polysaccharide resulted in reduction in disease due to group C strains in both the vaccinated and non-vaccinated children in the United Kingdom (31). However, the immune response against capsular polysaccharides is very specific and the selective pressure of the immune response combined with the efficient horizontal exchange by natural transformation in these pathogens can result in acquisition of new capsule types (32), which has led to an ever expanding number of capsule types in commercial vaccines.

The originally unexpected finding that parenteral administration of conjugate capsular vaccines affected colonization does not appear to apply to the protein antigens present in the protein-based vaccines against *N. meningitidis* (5), which is also observed in an experimental transgenic mouse colonization model (33). Notably we have observed that parenteral administration of meningococcal TbpB and factor H binding protein are both effective in preventing sepsis in mice by *N. meningitidis*, but a reduction of colonization is only achieved after immunization with TbpB (36). A potential explanation for this observation is that TbpB will always be expressed under the iron-limited conditions in the host while the expression of other surface proteins may only occur under specific conditions. Thus, the observed prevention of natural colonization by needle-free administration of a TbpB-based microparticle formulation in this study might also be dependent upon the protein antigen used, and we should careful not to overstate its potential application. Although the microparticle formulation was more effective at prevention of colonization than the Montanide Gel formulation (**Figure 5**, MPv1-TbpB vs Gel5-TbpB), the specific requirement for the components of the formulation (poly I:C, cationic peptide or polyphosphazine) has not been determined.

The Tf receptors likely arose in bacteria colonizing the upper respiratory tract of vertebrates over 320 million years ago (34) and provided selective pressures for sequence changes on Tf that underly the strict specificity of the Tf receptors for host Tf (35) that limit the host range of these bacteria. The ability to acquire iron directly from host Tf on the mucosal surface and within the body have resulted in these bacteria being responsible for important infections in humans and food production animals. As a consequence, there is considerable potential utility for this needle-free approach by just targeting the TbpB antigen. Thus, this study provides proof of concept for administration of vaccines targeting the human pathogens *N. meningitidis*, *Haemophilus influenzae* and *Moraxella catarrhalis*, that have been responsible for a substantial portion of meningitis, otitis media, sinusitis and lower respiratory tract infections in humans. Since the needle-free device is already in use for administration of anaesthetics in dental practice to replace needle delivery, it bodes well for potential administration of childhood vaccines directed against these bacterial pathogens.

In this study we administered the first immunization with the needle-free device at day 7 when substantial levels of maternal antibody were expected (**Figure 6****)** as it would potentially reduce the potential for development of Glässer’s disease during the period of waning antibody levels (from 4 to 6 weeks of life). We used a microparticle preparation (14) that demonstrated efficacy under these conditions. We anticipated that induction of a memory B-cell response might still be accomplished in the presence of significant levels of maternal antibody such that a substantial antibody response would be achieved after the second immunization. Notably, the titers of antibody at day 35 were substantial (**Figure 6**), confirming that a memory B-cell response was achieved during the first immunization. This result is quite interesting because it demonstrates that the primary phase of the specific immune response is not completely suppressed by passive anti-TbpB immunity and that the mere absence of an increase in IgG titers prior to the second immunization does not indicate suppression of the primary humoral response. The primary response may have occurred at low and undetectable levels in the presence of maternal antibodies transferred by colostrum. Thus, our vaccine formulation and immunization protocol were capable of eliciting an active humoral immune response adequate in covering the critical susceptibility window for *Glaesserella parasuis* infection (≤35 days old). This feature may clearly also be advantageous for the administration of vaccines in humans.

Unfortunately, this study was not able to provide strong experimental support for proposing the primary mechanism for prevention of colonization. This was partly due to the size of the study but also due to the complexity. The results presented in **Figure 5** demonstrate that the administration of the microparticle vaccine below the epithelial cell layer (Mpv1^(0/0)TbpB^ and Mpv2^(O/D)TbpB^) is associated with prevention of colonization, but that administration of the Montanide Gel formulations could also provide some protection. Deciphering the results of the oral and nasal secretory IgA and IgG levels (**Figure 6**) is complicated due to the unknown contribution by the natural colonizing bacteria and the lack of strong correlations of antibody titers with prevention of colonization. However, the results clearly illustrate that the TbpB antigen is a useful target for prevention of colonization, likely due to the fact that it will be expressed in the iron-limited environment of the host.

A unique attribute of this study is that its primary potential application will likely be in humans, but would normally have to be performed post-licensure, as the conventional small animal infection models are not suitable for this type of study. In spite of the clear advantages of a pig model regarding its anatomical features, it is challenging to secure funding to support this type of work for development of human vaccines. Our somewhat unique position of pursuing the development of vaccines for humans and food production animals in parallel enabled us to implement this preliminary study, but it may be challenging to secure additional funding from conventional government and academic sources. However, the proof of principle provided by this study and prior studies with pig pathogens (11–13) suggests that TbpB-based vaccines can be developed to provide a broadly cross-protective immune response capable of preventing colonization by porcine pathogens expressing any variant of TbpB, thus eliminating the reservoir for vaccine escape and infection. Effectively applying this proof of principle to *Neisseria meningitidis*, *Haemophilus influenzae*, *Moraxella catarrhalis* and perhaps even *N. gonorrhoeae* would have a substantial impact on human health thus hopefully will provide the opportunity to more fully develop this approach.

## Data Availability Statement

The raw data supporting the conclusions of this article will be made available by the authors, without undue reservation.

## Ethics Statement

The animal study was reviewed and approved by Health Sciences Animal Care Committee, Cumming School of Medicine, University of Calgary.

## Author Contributions

Conceived and designed the experiments: RF and AS. Performed the experiments: RF, SC R-hY, and GF. Contributed reagents/materials/analysis tools: AS. Wrote the paper: RF, SC, and AS. All authors contributed to the article and approved the submitted version.

## Funding

This study was supported by the Alberta Livestock Management Association (ALMA, 2016E012R). RF (sabbatical year) was financed by CAPES–Brazilian Federal Agency for Support and Evaluation of Graduate Education within the Ministry of Education of Brazil. SC was supported by a University of Calgary Eyes High Doctoral Recruitment Award.

## Conflict of Interest

GF was employed by AFK Imunotech. AS is a shareholder in Engineered Antigens Inc. but would derive no potential benefit from needle-free delivery of vaccines.The remaining authors declare that the research was conducted in the absence of any commercial or financial relationships that could be construed as a potential conflict of interest.
