# Supplementary material for: Proof of Concept for Prevention of Natural Colonization by Oral Needle-Free Administration of a Microparticle Vaccine
Source: Front Immunol. 2020 Oct 23;11:595320. doi: 10.3389/fimmu.2020.595320 (PMC7645216; doi:10.3389/fimmu.2020.595320)
Supplement: Supplementary file 1 [file DataSheet_1.docx]

SUPPLEMENTARY MATERIALS

## Supplementary Figures

**
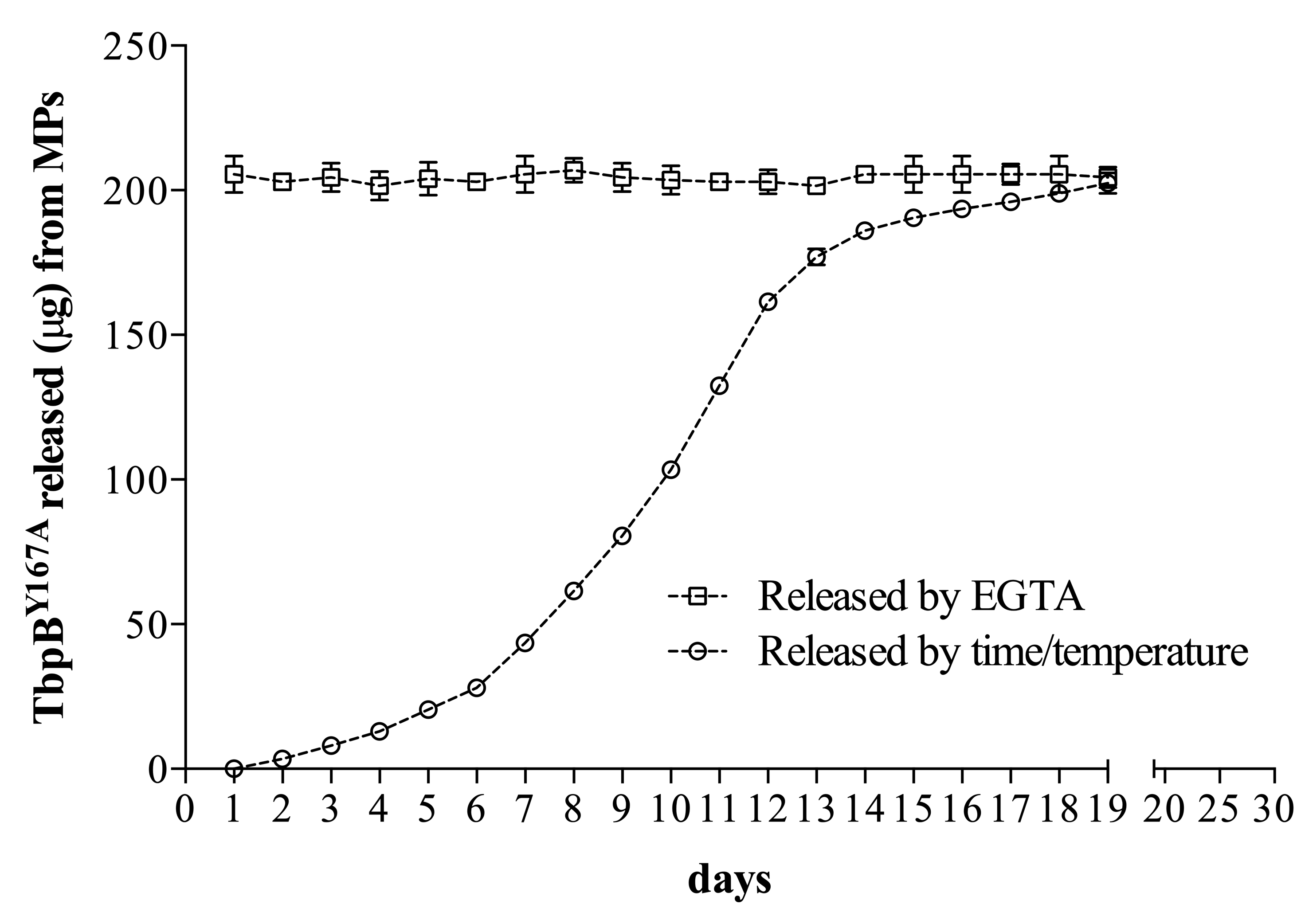
Supplementary Figure 1**. The release kinetics of TbpB from MPv-TbpB^Y167A^. MPs encapsulating TbpB^Y167A^ were incubated at 39ºC in DPBS. The protein released was assessed spectrophotometrically (absorbance at 280 nm) at various time points. The total amount of released TbpB of each single point is the mean of two different tubes assessed at that time point. The results were consistent in the 2 independent experiments conducted.

**
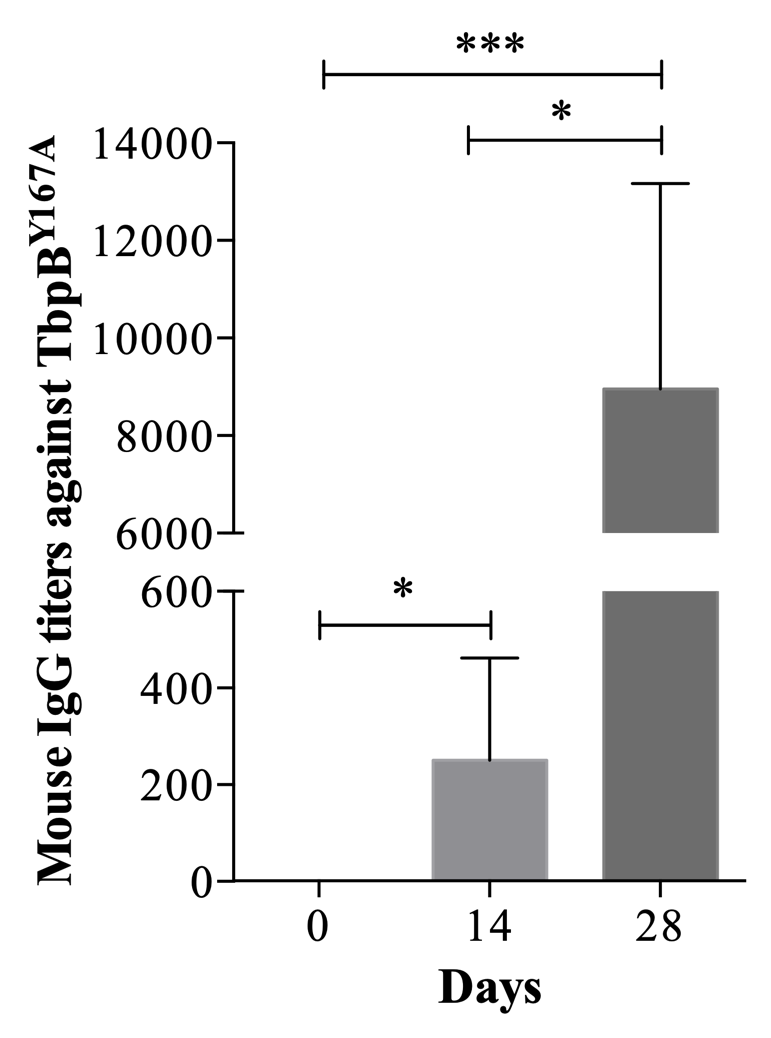
Supplementary Figure 2**. Kinetics of the specific antibody response in mice. Sera collected from different time periods during the immunization schedule were serially diluted and assessed by quantitative indirect ELISA based on TbpB^Y167A^ antigen. The titres (geometric mean + 95% CI) represent the reciprocal of the highest dilution with absorbance higher than two times of the absorbance values of the negative at 1:100 dilution.

Supplementary Tables

| **Findings** | **Groups and pig numbers** | | | | | | | | | | | |
| --- | --- | --- | --- | --- | --- | --- | --- | --- | --- | --- | --- | --- |
|  | **2×10^6^** | | | | **4×10^7^** | | | | **1×10^8^** | | | |
|  | **01** | **02** | **03** | **04** | **05** | **06** | **07** | **08** | **09** | **10** | **11** | **12** |
| *Death (dpc)* | 4^th^ | 7^th^ | 14^th^ | 14^th^ | 6^th^ | 6^th^ | 7^th^ | 14^th^ | 14^th^ | 14^th^ | 14^th^ | 14^th^ |
| *Pleuritis* | **+++** | **+** | Ø | + | + | **+** | **++** | **+** | **+** | **+** | Ø | Ø |
| *Pericarditis* | **+** | ++ | + | Ø | ++ | + | ++ | + | + | Ø | Ø | + |
| *Polyarthritis* | **++** | ++ | ++ | Ø | + | + | ++ | Ø | + | Ø | Ø | Ø |
| *Peritonitis* | + | + | ++ | Ø | ++ | + | ++ | + | + | + | Ø | + |
| *Meningitis* | **+** | Ø | Ø | Ø | + | + | + | Ø | Ø | Ø | Ø | Ø |

**Supplementary Table 1**. Intensity of pathological findings and date of death/euthanasia of individual pigs during the establishment of an intranasal infection model in conventional pigs. Ø – none; + – mild; ++ – moderate; +++ - accentuated.

| **Findings** | **Groups and pig numbers** | | | | | | | | | | | |
| --- | --- | --- | --- | --- | --- | --- | --- | --- | --- | --- | --- | --- |
|  | **2×10^6^** | | | | **4×10^7^** | | | | **1×10^8^** | | | |
|  | **01** | **02** | **03** | **04** | **05** | **06** | **07** | **08** | **09** | **10** | **11** | **12** |
| *Death (dpc)* | 4^th^ | 7^th^ | 14^th^ | 14^th^ | 6^th^ | 6^th^ | 7^th^ | 14^th^ | 14^th^ | 14^th^ | 14^th^ | 14^th^ |
| *Pleuritis* | **+++** | **+** | Ø | + | + | **+** | **++** | **+** | **+** | **+** | Ø | Ø |
| *Pericarditis* | **+** | ++ | + | Ø | ++ | + | ++ | + | + | Ø | Ø | + |
| *Polyarthritis* | **++** | ++ | ++ | Ø | + | + | ++ | Ø | + | Ø | Ø | Ø |
| *Peritonitis* | + | + | ++ | Ø | ++ | + | ++ | + | + | + | Ø | + |
| *Meningitis* | **+** | Ø | Ø | Ø | + | + | + | Ø | Ø | Ø | Ø | Ø |

**Supplementary Table 1**. Intensity of pathological findings and date of death/euthanasia of individual pigs during the establishment of an intranasal infection model in conventional pigs. Ø – none; + – mild; ++ – moderate; +++ - accentuated.

**
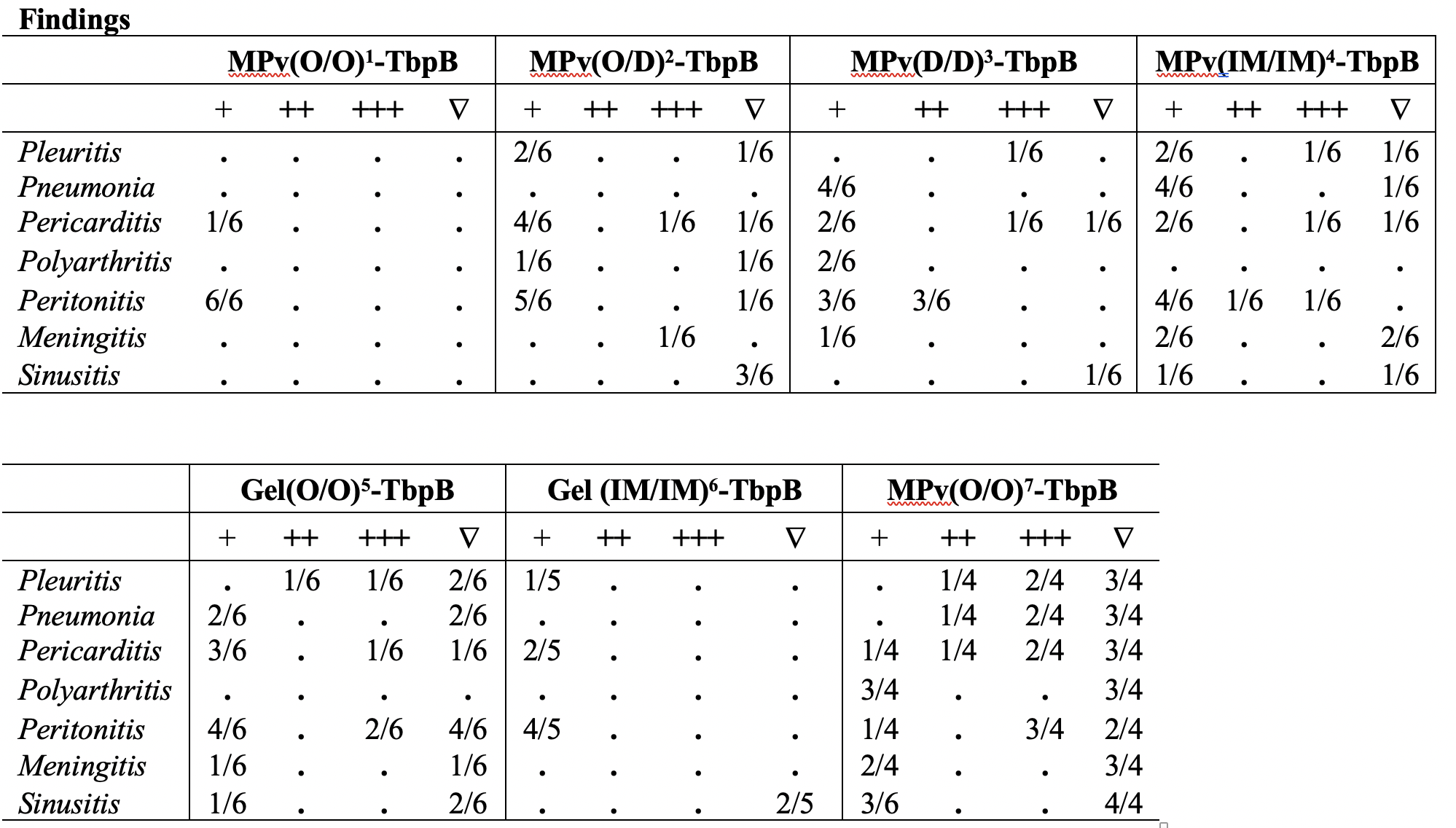
**

**Supplementary Table 2**. Main pathological findings and severity at necropsy of pigs vaccinated and challenged with *G. parasuis*. (+) mild; (++) moderate; (+++) severe; (∇) bacterial recovery from pig tissues.
